# Gamification in engineering education – An empirical assessment on learning and game performance

**DOI:** 10.1016/j.heliyon.2020.e04972

**Published:** 2020-09-18

**Authors:** Jenny Díaz-Ramírez

**Affiliations:** Universidad de Monterrey, Monterrey, N.L., Mexico

**Keywords:** Education, Gamification in education, Engineering courses, Motivation, Desired actions, Operation research, Higher education

## Abstract

Gamification, recently considered as a science, takes advantage of the benefits of games to induce desirable behaviors in a given “normal” activity. When applied in education, it is an approach to motivate and engage students in their learning process by incorporating game design principles. This paper presents the design and deployment of a game conducted in parallel with two groups of the engineering course “operations research”. The game design is theoretically supported, and unlike literature, the game proposed has the main distinguishing features: (a) it is carried out in parallel with the standard course, where player participation is optional, with extrinsic motivators regarding the final grade, (b) it lasts the entire semester, and it was applied to different groups with the same instructor, (c) in addition to academic performance, it has other social relatedness desired outcomes, (d) it combines the use of specific game elements divided in three types of activities: Mastery, related to the core topics of the class, Institutional, related to the university life and community, and Teamwork activities; and (e) it uses a WhatsApp chat group as the common communication platform. The assessment is twofold: the effects on learning, measured in two indicators, failure rate and average grade; and the perception of the game itself. Statistical results present empirical evidence of the positive effects of gamification on academic performance and other desired behaviors of social relatedness, such as a sense of belonging and teamwork.

## 1 Introduction

Gamification has been defined as “the use of game elements and game-design techniques in non-game contexts” (Deterding et al., 2011) or just “the process of making activities more game-like” (Werbach and Hunter, 2012). Also, it is defined as “the craft of deriving fun and engaging elements found typically in games and thoughtfully applying them to real-world or productive activities” (Chou, 2016), or similarly, as the process in which “one adds game elements to change a process that already exists to change how that process influences people” (Landers et al., 2018). When gamification is seen as a process, it involves the selection, application, implementation, and integration of game design elements, rather than simply their use (Werbach and Hunter, 2012) with the aim to “foster human motivation and performance in regard to a given activity” (Sailer et al., 2017). In summary, these definitions call for taking advantage of the benefits of games to induce desirable behaviors in a given “normal” activity.

The research trend on this topic shows an increasing number of empirical studies (Rodrigues et al., 2019). Based on game design theory, human behavior theories, and motivation theories (such as Self-determination theory (Ryan and Deci, 2000)), current research tries to justify and prove the causality effects between the introduction of game elements in normal activity and the desired change in a response variable, that reacts to the stimulus produced by these game elements.

Many game elements have been identified to be relevant to gamification. The most common ones are the triad: Points – Badges - Leaderboards, known as PBLs (Subhash and Cudney, 2018; Werbach and Hunter, 2012). Among many other game elements used in games as well in gamified non-game contexts are avatars, collection sets, countdown timers, levels, mentorship, narratives, status points, exchangeable points, progress bars, quests, trophy shelves, virtual goods, rewards, win-states, and yet this list is not exhaustive. It is argued that the specific game elements are the ones that have specific psychological effects, not the gamification process as a generic construct since it can take many forms and combine game elements in many different ways [4], so the focus of inquiry should be the impact of different game elements within a given context.

The success of gamification relies on the power of the motivation to induce desired actions. Based on this, Chou proposed a gamification design framework, called Octalysis, that includes 8 core drives, under the premise that “… if there are none of these Core Drives behind a desired action, there is no motivation, and no behavior happens” [2]. The Chou's core drives are:

The Chou's core drives are:

(1) Epic Meaning & Calling refers to when people believe they are doing something greater than themselves.
(2) joyDevelopment & Accomplishment is the drive for doing better, developing skills, and achieving mastery.
(3) Empowerment of Creativity & Feedback is expressed when users are engaged in a creative process.
(4) Ownership & Possession is where users are motivated because they feel like they own or control something.
(5) Social Influence & Relatedness incorporates all the social elements that motivate people
(6) Scarcity & Impatience is the drive of wanting something simply because it is difficult to reach.
(7) Unpredictability & Curiosity is the drive of being engaged because of the uncertainty of what comes next.
(8) Loss & Avoidance is the motivation to avoid something negative from happening.

Chou argues that its Octalysis approach covers the three elements of the Self Determination Theory (Ryan and Deci, 2000), which is a well-known approach to human motivation and personality. This theory states that autonomy, competence, and relatedness experiences foster high-quality forms of motivation and engagement.

Werbach and Chou (Chou, 2016; Werbach and Hunter, 2012) recommend that gamifying a productive activity should be human-focused, optional, structured, intrinsic, and fun; taking care of the performance objectives, the feedback, and the possible conflicts that may arise with the interaction with reality. Motivation can be extrinsic or intrinsic. The first one is derived from a goal, purpose, or reward, while the second one is simply the motivation you get by inherently enjoying the task itself. So, in game design, it is suggested to attract people into an experience with extrinsic rewards, and moving toward more intrinsic motivators to ensure a long term engagement.

### 1.1 Gamification science

Landers et al., in (Landers et al., 2018), situate gamification science as a subdiscipline of game science, and define it as: “a social scientific, post-positivist subdiscipline of game science that explores the various design techniques, and related concerns, that can be used to add game elements to existing real-world processes”, and argue that meaningful conclusions should be drawn based on human behavior, which is reactive to interventions. They propose a causal model supported in four types of person-focused constructs, as follows:

• Predictor Constructs, which are the initial causal force that created the change toward the desired outcomes (i.e. game elements).
• Criterion Constructs, which are the outcomes of interest.
• Mediator Constructs, which explain how gamification results in criterion change. A mediator is a variable that occurs causally between two others, in this case, the predictor and the criterion. There can be causal, direct, and indirect effects between them (predictor – mediator, mediator – criterion).
• Moderator Constructs, which are circumstances under which gamification is successful. A moderator is a variable that affects the direction and/or strength of the relationship between a predictor and outcome variable. (Baron and Kenny, 1986 in (Landers et al., 2018)).

Finally, Landers et al. argue that the impact analysis should be (and can be) done on specific game elements than by complete games, since it becomes impossible to conclude which game elements or interaction between them cause the observed changes.

### 1.2 Gamification in engineering education

“The importance of sustaining students’ motivation has been a long-standing challenge to education” (Dichev and Dicheva, 2017). Gamification in education refers to the introduction of game design elements and gameful experiences in the design of learning processes (Dichev and Dicheva, 2017). Seen as a form of active learning (García-Peñalvo et al., 2019), this is an approach to motivate and engage students in their learning process by incorporating game design principles, hoping that emotions such as fun, derived from games, can put the players in a “receptive frame of mind for learning” (Demkah and Bhargava, 2019). Gamification in higher education has received attention since 2013 and grew rapidly thereafter (Subhash and Cudney, 2018).

Only at Scopus database, for example, there are more studies in the last two years (i.e. since 2018) than ever before, with the Boolean phrase (Gamification AND education AND engineering AND assessment). The systematic review in (Subhash and Cudney, 2018) analyzed 41 out of 602 papers found in different databases with the keywords “gamification” AND “higher education”. Half of them dealt with engineering or science subjects. They found that among the main benefits of gamification observed in these studies are academic performance, engagement, motivation, and better attitude. However, according to (Dichev and Dicheva, 2017), by 2017, there was still insufficient evidence of high quality to support the long-term benefits of gamification in an educational context.

In addition, studies about the benefits of applying gamification in engineering courses are limited, mostly to ITC courses. The editorial paper (Minović et al., 2016), summarizes a special issue dealing with gamification experiences in the field of engineering education, with applications to product design, surgery skills, logistics, and other ITC applications. Mangeshkumar & Deepshikha (Demkah and Bhargava, 2019), tested a one-session gamified activity, on the performance of first-year electrical and electronics engineering students. Hakulinen et al. (2015), studied the effect of only one game element: achievement badges in a university-level computer science course. Though they found positive comments on motivation, there was little difference in the studied criteria: time management, carefulness, and learning. Faghihi et al. (2014), studied the effect of gamification of an app for Math learning, with a specific test, and compared it with other IT learning options. Statistically, no improvement in performance was found. Kasahara et al. (2019), studied the effects of leaderboards, a game element, in intrinsic motivation. They tested 35 students taking a C programming course at Waseda University for six weeks. They found students improved code metrics under gamification conditions without additional rewards, despite competition-related game elements, such as leaderboards, are not recommended in learning-focused environments (Chou, 2016). Juneyoung et al. (Park et al., 2019), studied the effects of rewards, another game mechanics, in the context of English vocabulary learning with a 3-day experiment with 64 students from a large-size university in Korea. They found that the performance-contingent rewards produced statistically significant learning enhancement compared to completion-contingent rewards.

Recently, in (Sánchez-Martín et al., 2020) authors studied the effects of a form of active learning gamified activity called “escape room” in more than 100 university students of four science classes. The objectives were to increase the student's motivation and to promote a positive experience, and to review the knowledge acquired during the other class activities. They found this activity as a successful tool for improving the acceptance of courses that are perceived as difficult, for building up a specific vision of the courses, and for creating positive emotional reactions toward the class. However, they didn't connect these results with performance in terms of knowledge content acquisition.

Other cases also study the effect of specific, short-term game-related activities in the academic performance of the students. In (Demkah and Bhargava, 2019) authors measure the effectiveness of gamification in learning, a class of 22 first-year engineering students in Electrical and Electronics was invited to participate in an activity-based learning session. They found a positive and more collaborative attitude, enjoy in the tasks assigned and better performance in the questionnaire applied. Based on the premise that time-on-task increases learning across all learning contexts, in (Landers and Landers, 2014) authors evaluated the effect of a gamified version of an online wiki-based project, by using a single game element: leaderboards. They used data from 109 students of an online upper-division industrial/organizational psychology course at a large U.S. east coast university. Results supported the basic premise and the relationship between the use of leaderboards and time-on-task as a mediator for the improvement in academic performance. In (Langendahl et al., 2016), authors analyzed three cases of real teaching situations in marketing and sustainable development disciplines in Sweden. They found these four game elements particularly useful to enhance student engagement and motivation: narrative, challenges, progression, and feedback. They also framed the experiences into cognitive, performative, and normative aspects of teaching and learning. From this framework, they concluded that gamification can be used to surprise and disrupt the students, to encourage them to be active in class, and to make learning fun. Later, inn (Sanchez et al., 2020), the effects of gamified quizzes in an educational setting were studied in two ways: if the gamification can enhance the testing effect, and if the student characteristics affect the effects of gamification. They conducted an experiment with data from two consecutive semesters of students enrolled in an introductory psychology course. They found that gamification did not enhance the testing effect and that student abilities can impact outcomes of gamification. However, the direction of causality in the last finding is assumed. Among their practical recommendations when including game elements in existing instructional content are (a) to avoid using the same game elements for long-term assignments, and (b) to take care of the different students' learning needs to permit lower-achieving students to also get benefit from them.

On the other hand, in (Zainuddin et al., 2020) the effects of gamified quizzes were also studied at the high school level. They found no significant differences in performance but they did in perceived motivation and positive attitude towards the learning activities. In addition, in (Hanus and Fox, 2015), authors studied the effects of a gamified Communication course at a large Midwestern university, with a final sample of 80. The longitudinal element claimed in this work is due to the assessment in three different moments of the same academic term (i.e. 16 weeks). Results suggest that some common game mechanics used in the classroom did not improve educational outcomes (grades) but on the contrary, they can harm motivation, satisfaction, and empowerment.

### 1.3 Gamifying the “operations research” course

Engineering courses in the middle of engineering programs (i.e. for sophomore and junior students) usually require the students to start thinking on applying the basis (mathematics and sciences courses) in real and more tangible contexts, and they face the need to further develop their problem-solving skills. Courses such as Operations Research (OR) is one of them. It is a core engineering course for industrial engineering-related programs, offered usually during the second year. It is usually perceived by the students as “difficult”, “interesting” and “challenging”. Unfortunately, the student's engagement is heavily motivated by stress, fear of failure, and anxiety, among other reasons that are related to the belief that their engineering bases are not so strong to be capable of succeeding in the subsequent courses. These aspects relate to Chou's drive cores #2: development and accomplishment, and #8: loss and avoidance. Extrinsic motivators work well on drive core #2, although there is an acknowledged risk that extrinsic motivators will outweigh the intrinsic motivation of deep and real learning.

Improvement of student's learning is difficult to measure, so the game is designed based on the causality model proposed in (Landers et al., 2018), and framed in the Chou's drive cores. It can be reflected in a more positive attitude towards the class, closing the learning loop (e.g. in the game players do not finish until solving completely and correctly the challenges), increased feedback (i.e. more players making specific questions, more office hours demanded), a higher level of challenges solved, and better grades, among others.

This work has a twofold purpose: it aims to measure the improvement of the learning process through the design, implementation, and assessment of game-like activities comprised in a game shape along with the entire academic term of an engineering course; and to assess the perception of the game itself. Unlike the literature, the game in this work is proposed to be carried out “in parallel” with the “standard” class, since the participation in the game is optional and “independent” of the class dynamics and policies. However, the final results in the game can have an impact on the final grade in the course.

In this work we assess the performance and effectiveness of the game through the following questions:

RQ1: Do the active players have a lower failure rate than non-active?
RQ2: Do the active players have better performance in the class (i.e. grades) than those non-active?
RQ3: Do the game dynamics that are proposed contribute to a better learning?
RQ4: Can the game foster a sense of belonging to the institution?
RQ5: Do the students like and enjoy playing the game?
RQ6: How are the game dynamics and their implementation perceived by the players?

## 2 Method

In an attempt of putting together the guidelines found in the literature for the design of a gamified activity that drives to desirable actions, we describe the gamification process of the OR course that was developed. First, the context in which the game was developed and the players (i.e. OR students) is presented. Then the game is described, including its elements and mechanics as well as the connections with Chou's core drives and the corresponding desirable actions. Finally, we describe the assessment instrument applied to the players at the end of the game and the course.

### 2.1 Players and context of the game

The game was applied to two OR groups during Fall 2019, offered to sophomore and junior students of industrial and systems engineering and management engineering programs of the Universidad de Monterrey, Mexico, with the same instructor, same rules, and same program content and grade policy. The players are young people, between 19 and 22 years old, with medium and high family income, students of a private university in the city. Students were invited to participate as players in a game by entering in a WhatsApp group-chat (WGC).

### 2.2 The game

In the game, players develop a series of activities of three different categories to obtain points along with the academic term, which is 16-week long. The activities are optional and rewards vary depending on their “difficulty level” or the “interest” of the designer (i.e. the professor). Three different kinds of badges can be earned, depending on the activities done; they are Mastery, Institutional, and Teamwork. Three different status levels (called: Shy, Regular, and OR Fan) could be achieved depending on the level, frequency, and type of the activities. Badges can be exchanged for benefits to the players, most of them related to or affecting the grades or convenience conditions in the class (i.e. possibility to choose the partner, the time for a presentation, the option of eliminating the worse grade, extra grade points in an activity, and even the option to exempt part of the final exam). The more a player participates, the easier it will be to win badges. The more a player participates, the more evidence of additional “home” work he/she will have; and therefore, more chance to better develop the competencies looked to be developed in the class.

#### 2.2.1 Initial considerations

Because of the potential heterogeneity in their mathematical basis, competitive approaches should be done carefully; and because of the “mathematical” focus of the class, challenges should favor both individual achievement and peer collaboration. Rewards are related to grades or favor the potential for higher grades. A better grade is by far the best external motivator in college students. In addition, participation in the game should be completely optional. This means that not subscribing as a player or any passive behavior in the game should not affect negatively the student's grade. In addition, The WhatsApp option is considered as a pilot of a future mobile App implementation.

#### 2.2.2 Activities

The game includes activities of three types: Mastery activities, related to the core and content of the class, College activities, related to institutional events, and Teamwork activities such as working with a different partner each time, helping others, etc. Mastery activities are available along the academic term. When the player finishes a challenge, the solution is shown and justified to the instructor, who accepts the challenge achievement or invites the player to come back with the corresponding corrections. The participation or assistance in institutional events and in teamwork challenges is confirmed by sending a picture, stamp or another doubtless evidence through the WGC. The activities are published periodically, together with the points to be granted and the due date. Once confirmed the achievement of the activity, the points are registered and published.

#### 2.2.3 Game mechanics

**Figure 1:**
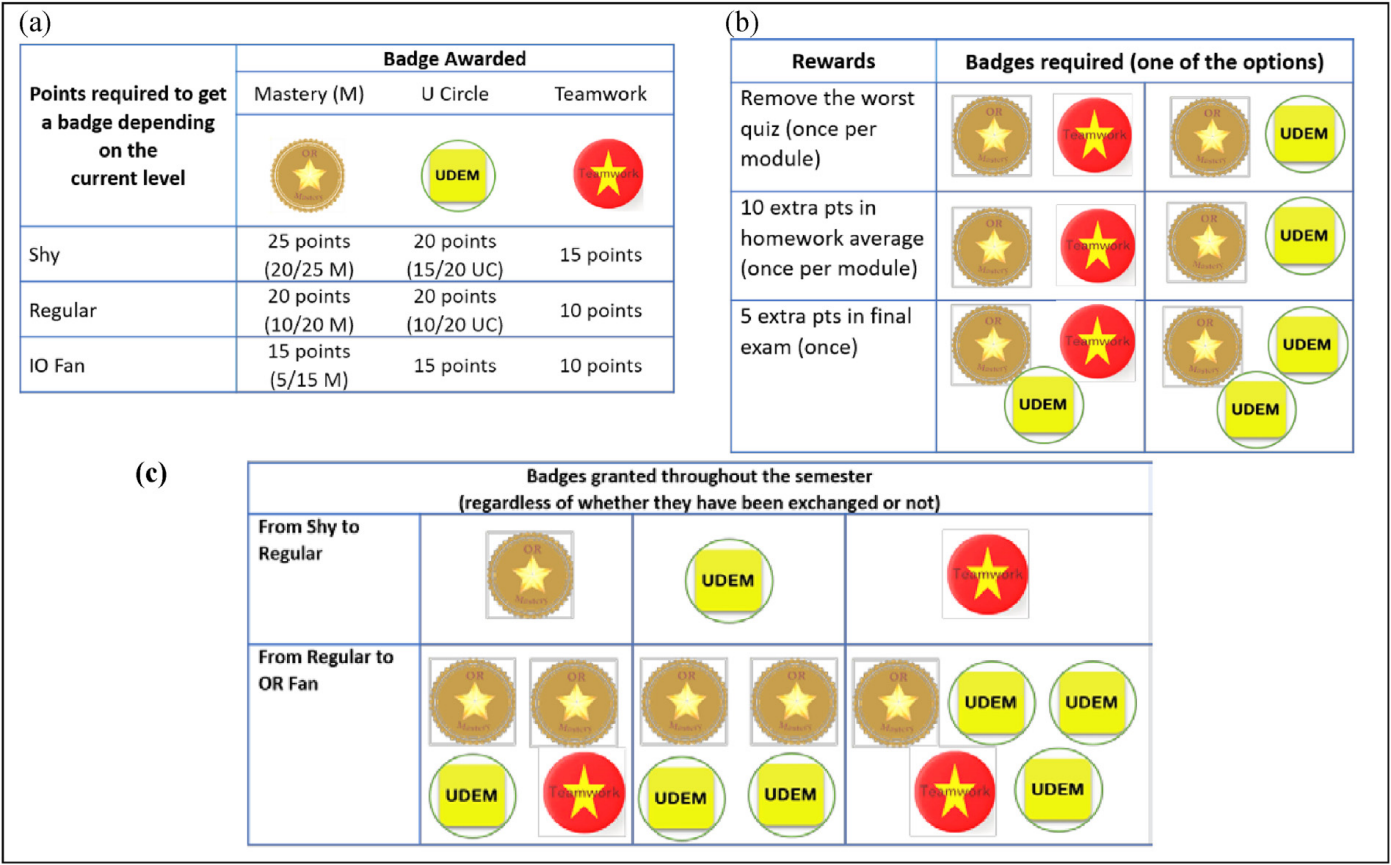
Some game mechanics used in the game: **(a)** Points required to get a badge. (e.g. a shy player can get a Mastery badge with 30 points, of which 20 are Mastery points), **(b)** Trading system example. **(c)** Leveling up scheme.

The points can be traded for badges, also of the three types: Mastery, Institutional, and Teamwork. Different combination of badges can be traded for benefits, such as: removal of worst quiz, extra points in exams, partial exoneration of final exam, options of choosing partners or schedules, etc. The list of the exchange possibilities is published from the beginning of the game in the WGC, and it was updated twice during the academic term. To keep the engagement along with the academic term, the game includes a leveling up strategy. Just for entering the game, some initial teamwork points are granted, and the player is at a “shy” level. Once the players make their first “transaction” for badges, they can level up to a “regular” level, and there will be a third “OR fan” level for the most active players. The higher the level, the “cheaper” or easier will be the trade for benefits. Any transaction for benefits requires the player trade Mastery badges, to foster additional “academic” work. Figure 1(a) shows examples of the tradeoffs, and Figure 1(b) shows the leveling up scheme.

Every reward to be granted during the game obeys to the validation with the instructor of the evidence by confirming that a challenge was achieved or the activity was done. The validation dynamics of extra activities directly related to the class, called “Mastery” activities, was personally done by the instructor, which favored the proper individual feedback. It also helped make sure students finish and solve correctly and completely many of the activities proposed during the game.

### 2.3 Causal relationship model

**Figure 2:**
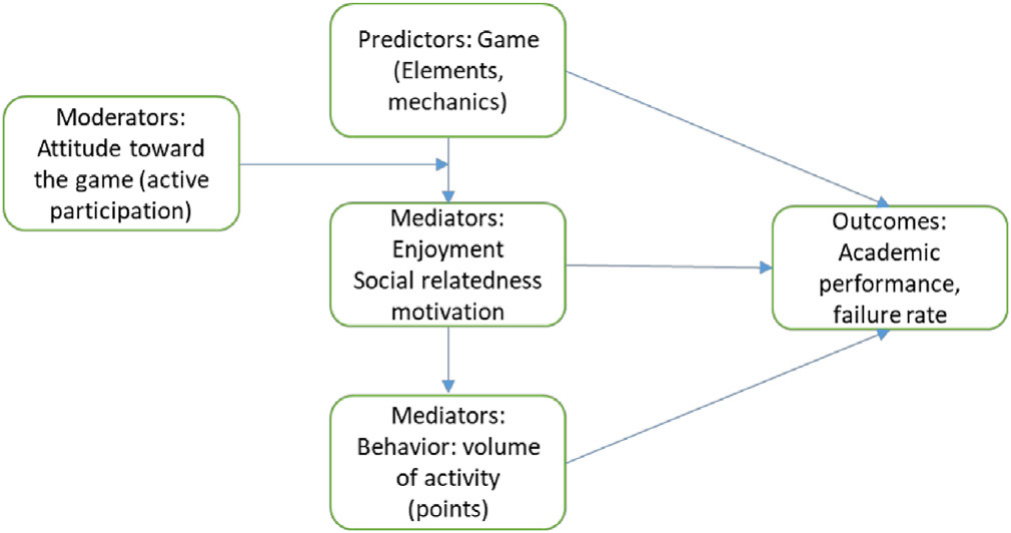
Causal relationship model.

Based Figure 2 shows The causal relationship model in Figure 2 schematizes how the different constructs of our game are related. It is based on the theoretical causal relationships model in gamification science by Landers et al. (2018), and it is consistent with the findings of the systematic review in (Rodrigues et al., 2019), that shows an indirect link between gamification and learning through the themes within the game concept: applications, design, and game.

### 2.4 Desirable actions

Table 1 describes the connections between the game elements used in the game, with the core drives and desirable actions (or outcomes in the causal model in Figure 2) towards the achievement of the game objectives.

**Table 1:** Connections between game elements, desirable actions, and metrics.

Game element	Core Drive	Desirable Action	Metrics∗
Mastery activities • Normal and higher difficulty level challenges • Assignments correction • More chances for players to do it better	(2) Development & accomplishment (4) Ownership and possession	Increased self-study and practice Pushing from completion to performance contingent activities. The chance to work on more interesting and challenging problems for the most active players	challenges counting points awarded benefits achieved M1-M5 indicators. G3
Institutional activities • Cultural activities • Academic activities • Official activities	(4) Ownership and possession (7) Unpredictability & Curiosity (5) Social influence & Relatedness	Sense of belonging – Community sense Social relationship	Student feedback: U1–U5 indicators
Teamwork activities • With in-class challenges • Mentoring activities • Work with a different partner each assignment	(5) Social influence & Relatedness (3) Empowerment of creativity & Feedback	A more positive attitude towards the class. To ask for help To work with others	Student feedback: T1-T5 indicators
Social interaction • Virtual rooms: WGC • Trading space: WGC	(1) Epic meaning & calling (5) Social influence & Relatedness (2) Development & Accomplishment	Social, freely and respectful interaction. Especially for low-performance students: to ask for help and explanation, to others besides the instructor.	Student feedback: G2, G4-G8 indicators
Feedback by instructor • Master activities check • Teams. • Cooperation: players explain and help them each other.	(3) Empowerment of Creativity & Feedback	Closing learning loop: Players making specific questions. Points for Mastery challenges are granted only until solved completely and correctly. More “actual” office hours.	challenges counting, office hours Student feedback: M3, G6, G9, T2,
Rewards • Related to grades∗ • Easer environment to fulfill goals • Some freedoms	(2) Development & accomplishment (3) Empowerment of Creativity & Feedback (6) Scarcity & Impatience	Better academic performance: Better grades, less failing rate	Average grade, Failing rate Player feedback: G1, G3-G5.
Progression feedback (game) • A record of activities done • A record of points achieved • A record of badges granted, traded, and available for trade A list of trading options	(2) Development & Accomplishment (5) Social influence & Relatedness	The option to compare their achievements (for those competitor players)	G2, G7, G12
Instead of a leader board, a list of the players with recent activity and news about badges or benefits granted to the players.	(2) Development & Accomplishment	To envision win-states	G7

∗ See Table 2 to consult the questions associated to each measured variable.

## 3 Game impact assessment

A survey was distributed among the players from the two OR groups at the end of the semester and when the game was finished. The Research Division at the University confirmed that the survey and this study complied with all regulations and was conducted in accordance with best practices in ethics of the University of Monterrey, having the approval and consent of participants involved in this research when submitting their responses.

The survey was divided into three main sections, the first one had the purpose of identifying the kind of player; this is, active or non-active. A second section dedicated to non-active players, with the objective of understanding the reasons for their passive involvement in the game. Finally, a third section addressed to active players, in order to answer the research questions.

### 3.1 Non-active players

Firstly, to identify whether the respondent is an active or non-active player, questions about the points granted in the three types of activities were registered. For the purpose of this study, an active player is the one that obtained enough points to get at least one badge. The open questions to non-active players were: N1: What are the reasons for being passive? N2: In retrospection, would have you liked to participate more actively? N3: What was missing? N4: Should the game be repeated? and space for suggestions.

### 3.2 Active players

For active players, Likert-scale assertions were used with the scale: 1: Completely disagree to 5: Completely agree. There was one section for each activity category and one section for the other game dynamics. Table 2 describes the questions for active players, grouped per section, where type is L for Likert-scale questions and M for multiple option questions. Other open questions were included to receive free feedback and recommendations for future improvements of the game.

**Table 2:** Questions for active players.

Section	Code	Type∗	Topic	Assertion
Mastery activities	M1	M	Motivation	What was the main reason that motivated you to do Mastery activities?
	M2	L	Better learning.	Mastery activities helped me learn the topics better.
	M3	L	Closing learning cycle	The feedback received when delivering Mastery activities was useful to close the learning cycle on the subject.
	M4	L	Amount	The number of Mastery activities available was sufficient and adequate.
	M5	L	Joyfulness	I enjoyed solving Mastery activities.
	M6	L	Socialization	Solving the activities Mastery contributed to interact more with my partners.
	G1	M	Effort-reward balance	The effort-points relationship of Mastery's activities was adequate.
Institutional activities	U1	M	Motivation	What was the main reason that motivated you to attend UDEM activities?
	U2	L	Belonging sense	The UDEM activities contributed to increasing my sense of belonging and UDEM identity.
	U3	L	Enrichment	The UDEM activities were enriching (social/cultural/academic).
	U4	L	Amount	The number of available UDEM activities was sufficient and adequate.
	U5	L	Joyfulness	I enjoyed attending the UDEM activities.
Teamwork activities	T1	M	Motivation	What was the main reason that motivated you to participate in Teamwork challenges?
	T2	L	Social skills	Teamwork activities helped improve my social and teamwork skills.
	T3	L	Learning	Teamwork activities contributed to increasing my learning of the subject.
	T4	L	Amount	The number of Teamwork challenges available was sufficient and adequate.
	T5	L	Joyfulness	I enjoyed participating in Teamwork challenges.
Game dynamics	G2	L	Chat	The WhatsApp® group chat was an appropriate strategy (in the absence of an application)
	G3	L	Motivating rewards	The points and awards were motivating enough to decide to participate actively.
	G4	L	Clear rewarding	The dynamic of obtaining points was clear and adequate.
	G5	L	Clear trading	The dynamic of exchange for benefits was clear.
	G6	L	Adequate trading	The dynamic of exchange for benefits was adequate.
	G7	L	Feedback	The feedback was adequate and timely enough.
	G8	L	Joyfulness	I enjoyed participating in the game.
	G9	L	Extra learning	I learned a little more than expected thanks to my active participation in the game.
	G10	L	Do it again	I think the game should be replicated in future semesters.
	G11	L	Social interaction	The game motivated discussion spaces with my colleagues about action strategies.
	G12	L	Periodical feedback	The periodic advances made by others motivated me to participate more actively.

∗ M: Multiple choice, L: Likert scale.

In addition to the survey, records of the points, badges and rewards granted were available for analysis, as well as the final grades obtained by the students at the end of the class.

## 4 Results and discussion

All students from both groups made part of the game and voluntarily entered to the WGC, this is 56 students. It means, they started to play the game. Most of them did it in the first week of the game, about two weeks after the academic term began. The rest of the group did it in the following two weeks. However, not all of the players were active ones. The self-perception of activeness was consistent with our active player definition. 59% were active players. 64% of the players answered the final survey (36 students). They were 70% of the active players and 62% of the non-active ones (15).

### 4.1 Non-active players

Those that did not actively participate in the game due to lack of time, this is 62.5% of non-active players, in retrospect, would have liked to participate more actively. The rest (37.5%) did not believe to need or benefit from the game. Two-thirds of them still would not have liked to participate more actively. For future implementations of the game, suggestions were related to reducing institutional activities or include only or mostly class-related activities. 12.5% of non-active players considered that the game should not be replicated in future semesters.

### 4.2 Comparison between class groups

With normality verified with Anderson-Darling test for institutional and teamwork categories, we tested the differences between the two groups in the number of points obtained in each category: Mastery, institutional and Teamwork. From Table 3, with the p_values of 0.52, 0.60, and 0.06, respectively, we concluded that there is no significant difference regarding the level of activity of the players during the entire game. Only Teamwork was slightly different because some of the activities in class gave points in this category, and the groups behaved differently within the class. Based on these results, the further analysis uses the entire sample without distinction of the group.

**Table 3:** Comparison of the level of activity (points granted) in the game between groups.

	Mastery		Institutional		Teamwork	
	Group 1	Group 2	Group 1	Group 2	Group 1	Group 2
P_value AD test∗	0.403	<0.005	0.098	0.142	0.163	0.168
N	15	23	15	23	15	23
Average	28.33	23.65	28.53	32.52	12.47	16.78
Std. deviation	20.93	22.25	20.74	25.95	5.34	8.33
DoF∗∗	36		34		36	
t statistic	0.6486		-0.5239		-1.9459	
p_value	0.5207		0.6038		0.0595	

∗ AD test for normality.

∗∗ DoF for degrees of freedom.

### 4.3 Impact on performance

Regarding the final grades (RQ2), we analyzed the value obtained by the student before any change due to the game. Normality was also verified with Anderson-Darling test, and F tests for difference of variances were also performed. According to these results, the corresponding t-tests were applied to test: (1) the difference between grades from both groups; (2) whether the active players’ grades were greater than those of non-active players in group 1, and (3) a similar test in group 2. Table 4 shows that grades from both groups are statistically the same, and in both groups, the active players achieved better grades than non-active players. The average differences observed in each group were 15 and 6 points, respectively.

**Table 4:** Comparison of the grades between groups and between active and non-active players.

	Grades All		Active vs Non Active Group 1		Active vs Non Active Group 2	
	Group 1	Group 2	Active	Non-Active	Active	Non-Active
p_value AD test∗	0.808	0.577	0.434	0.447	0.988	<0.005
p_value F test∗∗	0.045		0.461		0.0724	
N	23	29	15	8	20	9
Average	77.567	69.913	82.909	67.549	77.106	70.681
Std. deviation	12.931	18.375	10.765	10.857	8.866	5.339
DoF∗∗∗	53		14		24	
Test	H_1_: $\;\mu_{1}\neq \mu_{2}$		H_1_: $\;\mu_{1}>\mu_{2}$		H_1_: $\;\mu_{1}>\mu_{2}$	
t statistic	-1.813		3.241		2.412	
p_value	0.075		0.002		0.012	

∗ AD test for normality.

∗∗ F test for difference of variance.

∗∗∗ DoF for degrees of freedom.

Finally, the following performance results were obtained: Out of the students who failed the course 100% and 86% in both groups, respectively, were non-active (i.e. only one active player failed the course). Historically, 15–25% of the students failed the course.

### 4.4 Game effects

**Figure 3:**
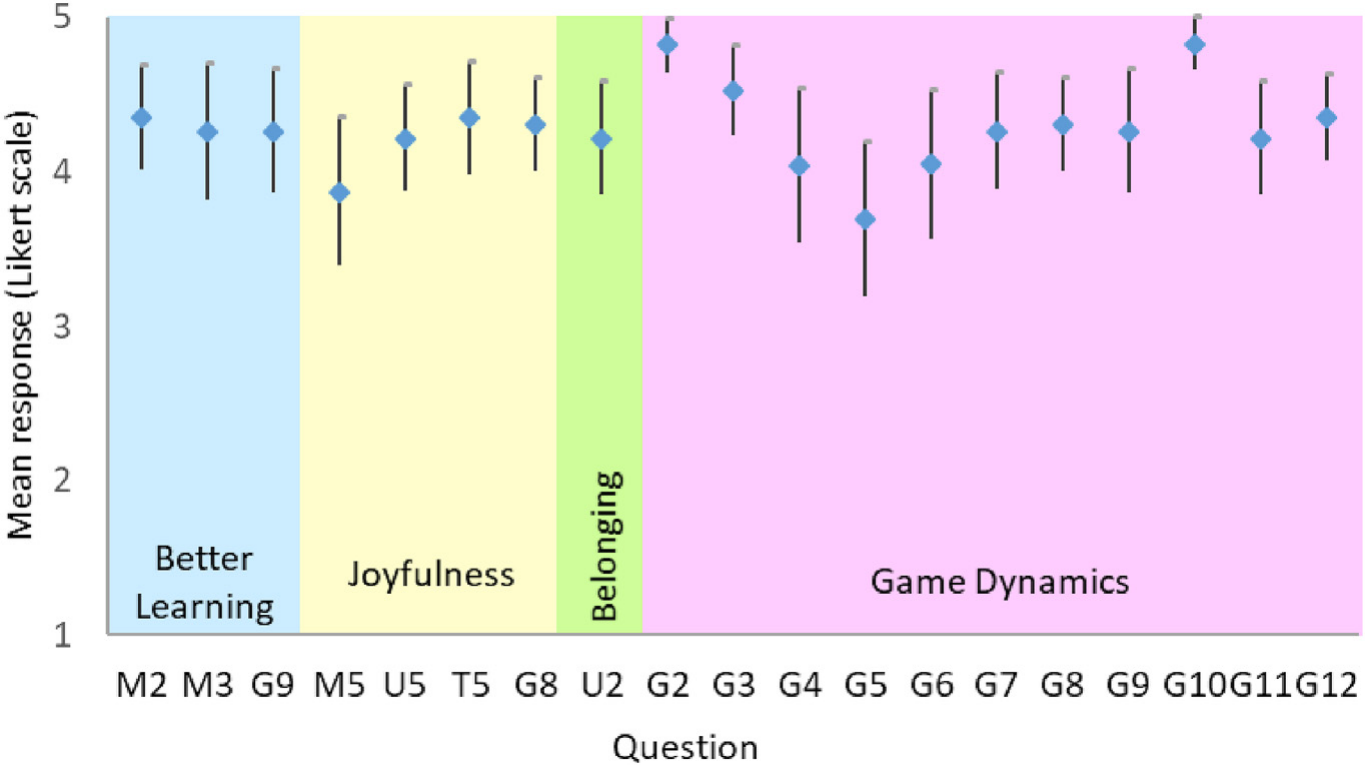
95% CI means on better learning, joyfulness, belonging sense, and game dynamics.

**Figure 4:**
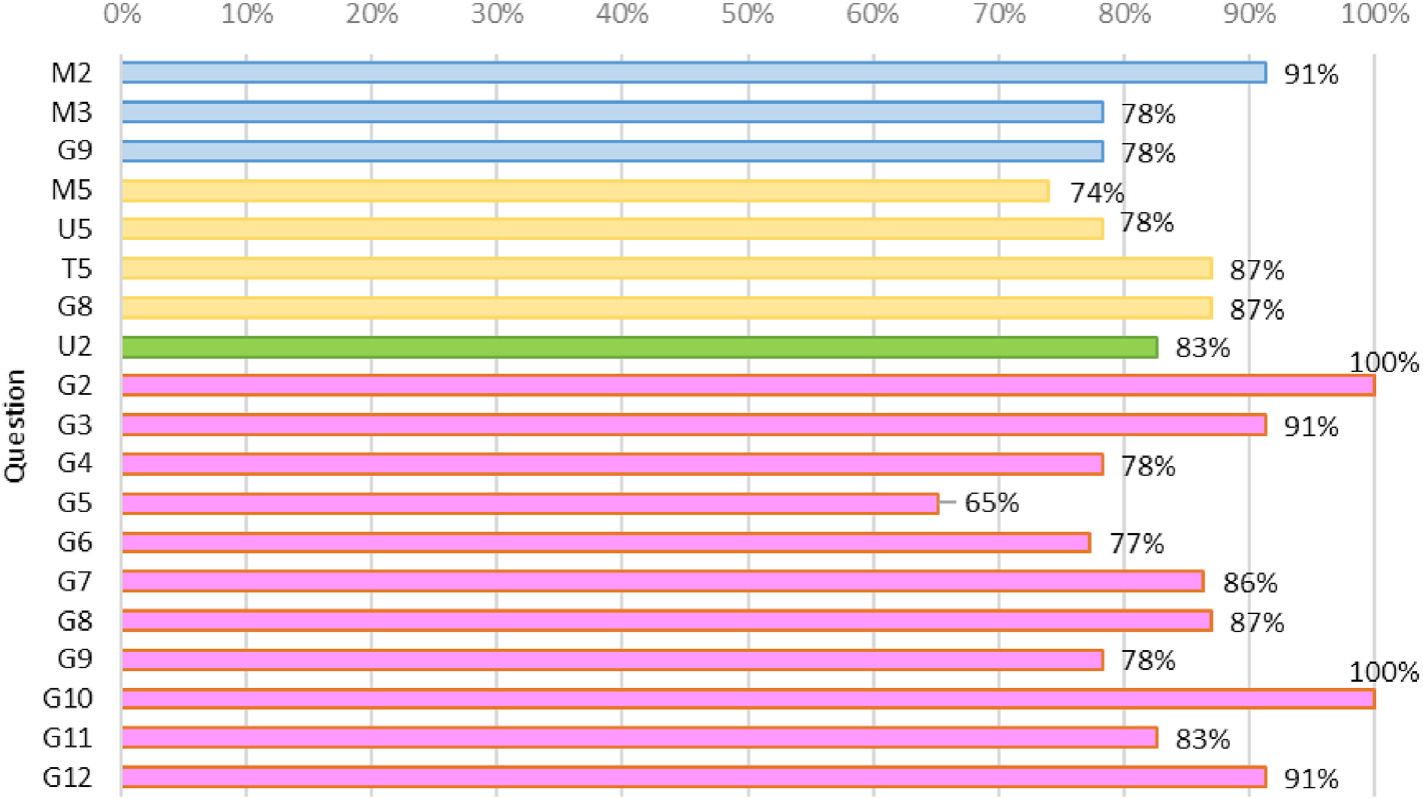
Proportion of positive answers.

The ultimate goal of the game is to be a tool that contributes to enhancing the students' learning. To assess the level of achievement of this goal (RQ3), three questions were analyzed: M2: better learning (mastery activities), M3: feedback to closing learning cycle and G9: extra learning (game). The Cronbach's alpha for this group of questions was 0.709 and for the first two questions was 0.8402, which gives a good sense of consistency about measuring the same concept: better learning. Figure 3 shows the 95% confidence interval means of the response to the questions in Likert scale and Figure 4 the proportion of positive answers; this is, with 4 or 5. . Statistical proportion tests were also performed and shown in Table 5 Values support the assertion that 90% of the active players believed they had better learning because of their participation in the game. A similar analysis was done to test if the game foster a sense of belonging to the university (RQ4) with question U2, and if the players enjoyed to play the game (RQ5) with questions M5, U5, T5, and G8. The Cronbach's alpha for this group was also acceptable (0.7184). Similar results on proportion tests are also shown in Table 5. Finally, to answer RQ5: “How are the game dynamics and their implementation perceived by the players?”, answers to the questions on game dynamics G2 to G12 were analyzed. With a Cronbach's alpha of 0.9080, we have the confidence of the consistency of the responses.

**Table 5:** p_values of hypothesis tests. H_0_: proportion of positive answers is 90%.

Section	Better learning			Joyfulness				Belonging
Question	M2	M3	G9	M5	U5	T5	G8	U2
p_value	1	0.162	0.162	0.111	0.162	0.723	0.723	0.281

Section	Game dynamics										
Question	G2	G3	G4	G5	G6	G7	G8	G9	G10	G11	G12
p_value	0.117	1	0.162	0.001	0.161	0.719	0.723	0.162	0.094	0.281	1

From these results, we can conclude that the chat on WhatsApp® was a successful strategy and that all players considered the game should be replicated. On the contrary, improvements in the communication of the exchange of badges and rewards are required. The remaining game dynamics passed the test of being well evaluated by 90% of the active players. In particular, 85% of the respondents thought the tradeoff between points and effort was appropriate, 82% that the WhatsApp option (WGC) was an appropriate strategy (in the absence of an application), and from 78% to 100% of respondents agreed with the positive assertions G4 to G10 (i.e. joyfulness, motivating rewards and feedback, social discussion, clear trading and feedback, future application, and extra learning); being clear trading the only aspect that did not passed the test of 90% of positive responses. Further discussion about the convenience, fairness and even the legality of the points-grades trading is worth considering as future work.

### 4.5 Game activities assessment

The Cronbach's alphas for the Likert questions from the three sections of the survey for active players; this is mastery, institutional, and teamwork sections, were 0.8749, 0.8276, and 0.8230, respectively. These values indicate that the questions in each section have relatively high internal consistency.

**Figure 5:**
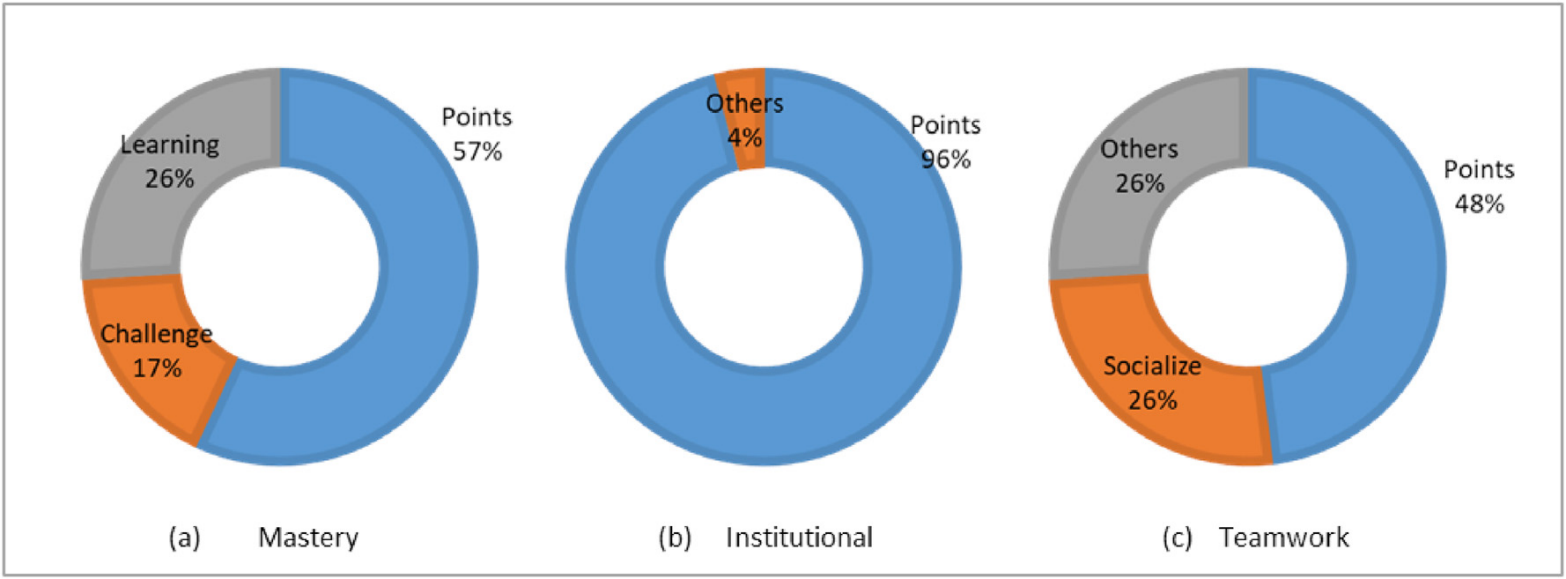
Main motivators per type of activity.

From Figure 5, we found that the predominant motivator for all of the activities was extrinsic: the points. However, intrinsic motivators for Mastery activities also worked for 43% of the sample. Finally, from Table 6 we observed that about three-fourths of the sample agreed with the positive assertions about feedback, enhanced teamwork, and joyfulness. 96% agreed that the number of Institutional activities proposed was appropriate; however, only 57% did it when regarding Teamwork activities, suggesting that further improvements (e.g. more options) would improve this perception. 83% of active players considered the Institutional activities included in the game were enriching and helped them to increase their sense of belonging to the institution.

**Table 6:** Summary of activities evaluation.

Activity	Better learning	Enhanced teamwork	Amount adequate	Joyful activities	Others
	% agreement (positive scores: 4 and 5)				
Mastery	M2: 91% M3: 85%	M4: 74%	M5: 70%	M6:74%	Feedback: M3: 78%
Institutional	-	-	U4: 96%	U5: 78%	Belonging U2: 83% Enriching U3: 83%
Teamwork	T2: 83%	T3: 78%	T4: 57%	T5: 87%	

## 5 Conclusions

This work contributes to the current study of the impact of gamification for better learning process in higher education, specifically in engineering courses, through the following aspects: (a) a game design process aimed to accompany the course, based on a causal model with specific desired actions, (b) a two-fold assessment procedure, first to assess the relationship of the game elements with desired actions and academic results (i.e. grades and perceived learning), and second, to assess the game itself (i.e. game elements perception), and (c) it presents empirical evidence of the positive effects of gamification on academic performance and other desired behaviors of social relatedness, such as a sense of community and teamwork.

A game accompanying the entire course of operations research offered to undergraduate engineering students in a Mexican university was designed and deployed to two groups with the same instructor. The game was designed following the structured models from (Chou, 2016; Landers et al., 2018; Werbach and Hunter, 2012) which are based on motivation theory and gamification experience. Activities related to mastering the class, sense of belonging to the institution, and teamwork, were the center of the game. Set it over a simple WhatsApp® group, with no narrative but with strong extrinsic motivators associated to the class grades, more than two-thirds of the students actively participated.

Results showed that students who actively played the game showed a substantial greater passing rate compared to non-active players, and high engagement in problem-solving activities, even of a higher level than the class. Active players perceived that the game contributed to a better learning process, the Institutional activities helped them to increase the sense of belonging to the institution, and in addition to the Mastery activities, the Teamwork activities helped to improve their learning. Furthermore, the extrinsic rewards were by far the main motivators that boosted active playing the game. Results also showed that improvements should be made in terms of joyfulness, feedback, and amount of Teamwork activities, as well as the inclusion of elements to promote more players be “active” since the first phases of the player journey.

## Declarations

### Author contribution statement

J. Díaz-Ramírez: Conceived and designed the experiments; Performed the experiments; Analyzed and interpreted the data; Contributed reagents, materials, analysis tools or data; Wrote the paper.

### Funding statement

This research did not receive any specific grant from funding agencies in the public, commercial, or not-for-profit sectors.

### Competing interest statement

The authors declare no conflict of interest.

### Additional information

No additional information is available for this paper.
